# Nanomedicine therapies modulating Macrophage Dysfunction: a potential strategy to attenuate Cytokine Storms in severe infections

**DOI:** 10.7150/thno.47982

**Published:** 2020-07-25

**Authors:** Jingping Liu, Meihua Wan, Christopher J. Lyon, Tony Y. Hu

**Affiliations:** 1 Key Laboratory of Transplant Engineering and Immunology, National Clinical Research Center for Geriatrics, West China Hospital, Sichuan University, Chengdu, 610041, China.; 2 Department of Integrated Traditional Chinese and Western Medicine, West China Hospital of Sichuan University, Chengdu, 610041, China.; 3 Center of Cellular and Molecular Diagnosis, Tulane University School of Medicine, 1430 Tulane Ave. New Orleans, LA 70112, USA.; 4 Department of Biochemistry & Molecular Biology, Tulane University School of Medicine, 1430 Tulane Ave. New Orleans, LA 70112, USA.

**Keywords:** cytokine storm, pro-inflammatory disease, macrophage dysfunction, nanomedicine

## Abstract

Cytokine storms, defined by the dysregulated and excessive production of multiple pro-inflammatory cytokines, are closely associated with the pathology and mortality of several infectious diseases, including coronavirus disease 2019 (COVID-19). Effective therapies are urgently needed to block the development of cytokine storms to improve patient outcomes, but approaches that target individual cytokines may have limited effect due to the number of cytokines involved in this process. Dysfunctional macrophages appear to play an essential role in cytokine storm development, and therapeutic interventions that target these cells may be a more feasible approach than targeting specific cytokines. Nanomedicine-based therapeutics that target macrophages have recently been shown to reduce cytokine production in animal models of diseases that are associated with excessive proinflammatory responses. In this mini-review, we summarize important studies and discuss how macrophage-targeted nanomedicines can be employed to attenuate cytokine storms and their associated pathological effects to improve outcomes in patients with severe infections or other conditions associated with excessive pro-inflammatory responses. We also discuss engineering approaches that can improve nanocarriers targeting efficiency to macrophages, and key issues should be considered before initiating such studies.

## Introduction

Circulating levels of pro-inflammatory cytokines correlate with the severity of many infections. Severe infections can result in uncontrolled immune responses accompanied by excessive release of pro-inflammatory cytokines, frequently referred to as a “cytokine storm”, that can be responsible for a significant degree of the pathology and mortality associated with severe infections 1. For example, excessive cytokine release appears to be a common feature of coronavirus disease 2019 (COVID-19), especially in severe cases, where it can produce acute respiratory distress syndrome (ARDS) to aggravate lung pathology, and sometimes progress to cause multiple organ dysfunction and failure 2-4. Excessive cytokine production in response to SARS-CoV-2 infection promotes apoptosis of pulmonary endothelial and epithelial cells to damage the microvasculature and cause vascular leakage and alveolar leakage responsible for ARDS pathology 5. Dysfunctional immune responses and excessive cytokine responses associated with COVID-19 appear to be influenced by several risk factors and underlying conditions 5, but our understanding of the factors that influence these responses in COVID-19, and other diseases subject to this pathological response, is still incomplete.

Effective therapeutic strategies to prevent or suppress cytokine storms and attenuate their effects, or other conditions prone to this response, during severe infection do not yet exist 6. This is partially due to limited understanding of the factors that regulate this process and what effect individual cytokines play in specific pathological responses 5. Clinical trials of therapeutics that target different cytokines associated with specific pathologies have recently been proposed for various diseases 7, but it is not clear if suppression of a single cytokine target is sufficient to attenuate pathologies related to cytokine storms, since individual cytokines may have distinct or synergistic effects in these processes. Approaches that instead target mechanisms resulting in the immune dysfunction responsible for excessive cytokine production may be a more feasible means to limit pathology associated with cytokine storms.

Macrophages and related monocyte-lineage immune cells, which reside in most body tissues and play essential roles in regulating cytokine-mediated inflammatory responses and innate and adaptive immune reactions, are good potential targets for such strategies 8-10. Circulating monocytes are recruited to injured or infected sites, where they secrete factors that can promote leukocyte invasion and activation. Normally, macrophage-mediated pro-inflammatory responses to injury or infection are self-limiting to allow pathogen clearance while minimizing tissue injury and remodeling. However, macrophage dysregulation during such responses can induce excessive cytokine production to promote local and systemic inflammation and injury. Excessive pro-inflammatory responses to SARS-CoV-2 infection are a major determinant of disease severity, and patients with severe COVID-19 cases exhibit cytokine profiles similar to those detected in macrophage activation-like syndrome (MALS), which is linked with macrophage dysfunction 11. Patients with severe COVID-19 also exhibit alveolar macrophage depletion, increased pulmonary invasion by pro-inflammatory macrophages, and elevated systemic numbers of IL-6-producing monocytes and macrophages 11. Macrophage dysfunction may therefore be a promising therapeutic target to inhibit cytokine storm syndrome in severe infections. Conventional small molecular inhibitors usually lack the ability to target distinct cell types or cell lineages, which could be necessary to achieve therapeutic drug concentrations while avoiding significant off-target effects. However, nanomedicine approaches employing nanoparticles designed to recognize cell-specific or cell-selective factors on target cells have the potential to address these issues to attenuate runaway immune reactions leading to cytokine storms 12. Similar nanotherapeutic strategies have been considered to improve intranasal drug delivery for potential COVID-19 therapies by employing the special properties of various nanomaterials to enhance their persistence in the mucosal environment, and subsequent cellular uptake, to increase therapeutic bioavailability 13.

In this mini-review, we focus on the pathology of cytokine storms in severe infections, highlight the role of macrophage dysfunction in this process, and discuss nanomedicine-based therapies that could be used to target macrophage dysfunction to improve clinical outcomes in patients with severe acute infections.

## Cytokine storms in infectious disease

### The pathology of cytokine storms

Many cytokines released during pro-inflammatory immune responses can activate specific leukocyte populations and target their recruitment to sites of inflammation *via* chemotaxis in response to concentration gradients 14. These cytokines can be divided into interleukins, chemokines, interferons, and growth factors 1, and the roles of tumor necrosis factor alpha (TNF-α), interleukin (IL)-6, IL-8 and members of the IL-1 family have been well studied in multiple pro-inflammatory responses 15. Cytokines play essentials role throughout inflammatory processes by promoting pathogen recognition, immune cell recruitment to sites of infection, threat elimination, and return to homeostasis 16. TNF-α and IL-1β can promote vasodilation and vascular permeability to enhance leukocyte infiltration responses, while IL-6 can stimulate the expression of complement proteins that play a key role in innate immune responses 15. Study of the role of specific cytokines and their receptors in specific pro-inflammatory processes can be challenging, and has implications for therapeutic strategies, since some chemokine receptors are reported to bind multiple chemokine ligands, leaving the chemokine network with a significant level of redundancy 17.

The term cytokine storm, first used in 1993 in reference to runaway inflammatory responses in graft-versus-host disease 18, has been widely adopted to describe a situation in which cytokines are excessively released in response to various disease or injury conditions. Cytokine secretion is an intrinsic part of the activation and regulation of innate and adaptive immune responses stimulated by pathogen exposure, but only a minority of pathogens, including some gram-negative bacteria and some viruses, such as SARS-CoV-2, routinely trigger cytokine storms, particularly ones sufficient to induce systematic organ damage and promote mortality. Events involved in cytokine storm initiation and progression are complex and involve multiple factors and cell types. For example, at the early stage of SARS-CoV infection, rapid virus replication induces a delayed release of interferons (IFNs) but increases the secretion of several pro-inflammatory cytokines, including TNF-α, IL-6, and IL-1β, which is accompanied by an influx of macrophages to the lungs. Activation of these macrophages through IFN signaling induces them to secrete chemokines (*e.g.*, CCL2 and CCL3) that promote local accumulation of other pro-inflammatory immune cells, including neutrophils, monocytes, and dendritic cells that can release more cytokines and reactive oxygen species to increase the severity of lung injury 5, 7, 19, 20.

Most individuals are vulnerable to SARS-CoV-2 infection, but can exhibit a broad range of symptom severity, and several risk factors are associated with cytokine storm development, including age (> 60 years old), smoking, poor nutrition and pre-existing conditions that can affect immune responses, including obesity, diabetes, hypertension, cardiovascular and chronic lung disease, and cancer 21, 22.

No specific therapeutic strategies have yet been developed to attenuate cytokine storm mediated pathology, despite the established link between cytokine storms and disease pathology and mortality.

### Consequences of cytokine storm in severe infections

COVID-19 offers a compelling example of the effect of cytokine storms on pathology and mortality. The precise mechanisms responsible for the pathological changes observed in severe COVID-19 cases are not well understood, but several studies have reported that most patients with severe pathology have markedly elevated circulating levels of multiple pro-inflammatory cytokines, including IL-1, IL-6, IL-8, IL-21, TNF-α, and MCP-1 2, 23. Cytokine storms are considered the primary cause of increased mortality in severe COVID-19 cases, since elevated levels of these cytokines can lead to ARDS, shock, and multiple organ damage and failure 6. ARDS is the primary pathology and leading cause of death in patients with severe SARS-CoV-2, SARS-CoV or MERS-CoV infections, but is also responsible for morbidity and mortality in patients affected by sepsis, pneumonia and other infections where cytokine storms play central roles in disease pathology 23. ARDS is characterized by an acute inflammatory response in the lung parenchyma that is associated with severe injury to the epithelial and endothelial barriers. Cytokines play a critical role as signaling molecules that initiate, amplify, and perpetuate inflammatory responses during ARDS development 5. Immunosuppression has thus been proposed as a means to resolve cytokine storms in severe COVID-19 cases, and clinical trials employing immunosuppressive drugs (methylprednisolone, hydroxychloroquine, chloroquine, and leflunomide); inhibitors of pro-inflammatory cytokines (IFN-γ, IL-1β, IL-6, IL-17A, M-CSF, and TNF-α); and modulators of factors that regulate innate and adaptive immune reactions (C5, CD47, GM-CSF, M-CSF, and sphingosine-1-phosphate) in COVID-19 patients are currently underway 7, 11. However, the immunomodulatory effect of targeting one or two cytokines may not be sufficient to attenuate cytokine storms, and their resulting pathology. It may instead be more effective to employ therapeutic approaches that target key immune cells that mediate cytokine storm development to treat severe COVID-19 cases.

## Role of macrophage dysfunction in cytokine storm development

### Role of macrophages in infection control

Macrophages are multifunctional and heterogeneous innate immune cells that reside in most tissues and play essential roles in innate immunity, the regulation of adaptive immunity, and pro-inflammatory responses to control the virulence and pathology of infectious diseases 8, 24, 25. During an infection response, tissue resident macrophages, and monocyte-derived macrophages recruited from the circulation, migrate to injured or infected sites to exert regulatory effects on the developing immune response 24.

Macrophages act as an initial defense against infection 24, 26-28 and are equipped with pattern recognition receptors (PRRs) that recognize pathogen associated molecular patterns (PAMPs) 28 to promote pro-inflammatory responses and pathogen engulfment and phagolysosome-mediated digestion 26. Mature macrophages can alter their phenotypes and undergo functional polarization in response to signals from the local microenvironment that induce naïve or resting “M0 macrophages” to adopt classically activated “M1-like” or anti-inflammatory “M2-like” phenotypes or promote M1-like and M2-like macrophage interconversion. M1-like macrophages produce elevated levels of reactive oxygen species to promote the destruction of engulfed pathogens, and secreted elevated levels of pro-inflammatory cytokines, such as IL-1β, IL-6, and TNF-α, to regulate local tissue and immune responses that promote pathogen clearance 24. M2-like macrophages, or “alternately activated” macrophages, exhibit more diverse phenotypes and are primarily involved in tissue repair and the resolution of inflammatory responses 25. However, macrophages can also develop mixed phenotypes in response to their local microenvironment rather than adopting extreme M1-like or M2-like phenotypes identified by *in vitro* characterization studies 8.

Macrophages can also display pathogen-derived proteolytic peptides on their type I and type II major histocompatibility complexes (MHCI and MHCII) to activate T lymphocytes that recognize these peptides and initiate a pathogen-specific adaptive immune response 27.

### Macrophage dysfunction and cytokine release during severe infection

A rapid and well-coordinated innate immune response is the first line of defense against infection, but excessive immune responses can cause local and systemic tissue dysfunction and damage 29, while weak immune responses can allow unchecked microbial or viral proliferation and resulting pathology. Phagocytosis of pathogens by macrophages can activate a type I interferon (IFN) response to promote adaptive immunity 8, 24 and stimulate pro-inflammatory M1-like macrophage differentiation and the secretion of several pro-inflammatory cytokines, including TNF-α, IL-1, IL-6, IL-8, and IL-12 30. These cytokines have local effects to increase vascular permeability and lymphocyte recruitment, and systemic effects to induce fever and the release of acute phase proteins 30. This response is beneficial when cytokines are released at appropriate levels, but harmful when cytokine secretion is deregulated or excessive. For example, elevated serum levels of IL-6, a hallmark of severe MERS-CoV and SARS-CoV-2 infections 4, 31, correlate with ARDS, respiratory failure, and adverse clinical outcomes in COVID-19 patients 2, 32. Monocytes and macrophages are the primary source of IL-6, which has prominent pro-inflammatory properties through its effect to activate Janus kinase (JAK) and signal transducer and activator of transcription 3 (STAT3) pathways, and can trigger cytokine production by T lymphocytes and neutrophils 33.

**Figure 1:**
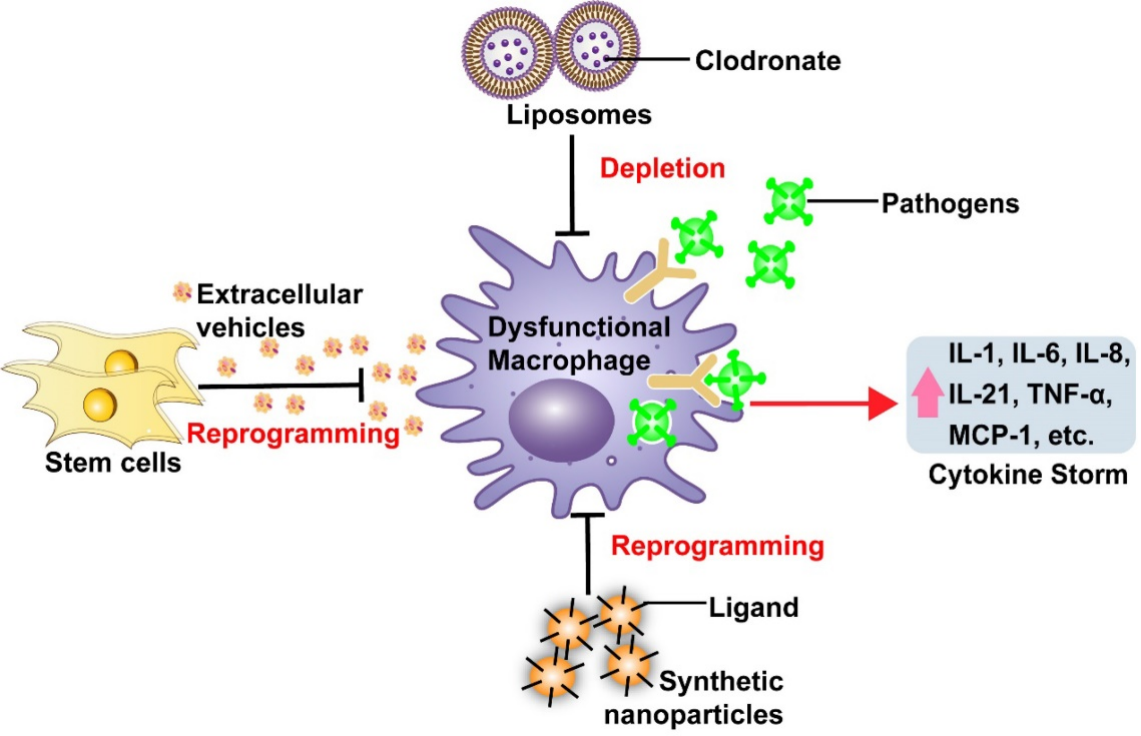
Effects of macrophage dysfunction to simulate excessive pro-inflammatory cytokine production during severe infection, and nanomedicine-based therapeutic strategies to counter macrophage dysfunction.

Macrophage dysfunction can initiate uncontrolled cytokine release leading to the development of cytokine storms observed in many severe infectious diseases, including SARS and COVID-19 (**Figure 1**) 4, 31, 34. Autopsy and necropsy studies have detected the accumulation of inflammatory monocyte-macrophages (IMMs) and neutrophils in the lungs of humans and animals following SARS-CoV infection 34. Patients who die from SARS exhibit extensive macrophage infiltration in alveoli and interstitial lung tissue, while the severity of lung lesions in MERS cases has been reported to correlate with the extent of macrophage and neutrophil infiltration, and the abundance of these cells in the peripheral circulation 34. More recent analyses indicate that large numbers of macrophages localize to the alveolar lumen of patients who die from COVID-19 35. These reports suggest that macrophages may be the primary source of cytokines and chemokines associated with lethal virus infections. Macrophages may also serve as viral targets and reservoirs to promote both virus replication and dissemination 36. Macrophages express angiotensin-converting enzyme 2 (ACE2) 37, the primary receptor for SARS-CoV and SARS-CoV-2, and their infection may increase their pro-inflammatory phenotype and response. SARS-CoV virus particles and RNA have been detected in macrophages and lymphocytes 34, and SARS-CoV-infected macrophages exhibit delayed but elevated levels of IFN and pro-inflammatory cytokine expression 34. ACE2^+^ macrophages containing SARS-CoV-2 nucleoprotein, which exhibit IL-6 up-regulation, have also been detected in the spleen and lymph node marginal sinuses of patients who died from COVID-19 38.

Macrophage dysfunction also plays important roles in the pathology of other infections caused by viral and microbial pathogens, particularly when these infections progress to sepsis, due to overlap of monocyte/macrophage responses to these pathogens. Macrophages are normally activated in response to recognition of danger-associated stimuli produced upon recognition of various components of viral and bacterial pathogens (PAMPs) *via* PRRs on or within macrophages, including several members of the Toll-like receptor (TLR) family 39. TLR signaling in response to viral and microbial pathogens can induce macrophages to undergo polarization to a pro-inflammatory M1-like phenotype and secrete factors that promote innate and adaptive immune responses, but excessive activation can produce a dysregulated macrophage response that can lead to local or systemic injury. Sepsis, a leading cause of death among hospitalized patients, occurs when host-derived factors released into the circulation during bacterial or viral infection cause systemic inflammation, which can lead to the development of tissue injury and organ failure. Macrophage dysfunction (also known as macrophage activation-like syndrome), and its association with the cytokine storm syndrome, is recognized as a major cause of the high mortality rate of sepsis 29, 40. Macrophage dysfunction is thus a key contributor for excessive inflammation and high mortality during severe infections and sepsis.

## Therapeutic approaches to modulate macrophage dysfunction

Both clinical and experimental findings strongly suggest that dysfunctional macrophages are a major source of inflammatory cytokines in severe infections, and thus therapeutic interventions that target these macrophages may prove beneficial in attenuating cytokine storms that contribute to the increased pathology and mortality of severe infections. Therapeutic approaches designed to manipulate macrophage responses using conventional drug delivery mechanisms have been studied in preclinical models of inflammatory diseases and cancer 41, 42. These can be grouped into three strategies: (i) depleting macrophages to reduce dysregulated macrophage activity *via* therapies that exhibit macrophage-specific or -selective toxicity; (ii) inhibiting macrophage invasion to limit inflammatory responses at disease sites by blocking chemotactic monocyte surface receptors (*e.g.*, CC-chemokine receptor 2 (CCR2) 43; or (iii) reprogramming macrophages via interventions that employ anti-inflammatory factors or cytokine inhibitors to attenuate pathogenic macrophage phenotypes 44. However, while treatment with macrophage-depleting drugs, such as colony-stimulating factor 1 receptor (CSF1R) inhibitors and clodronate, or with CCR2-CCL2 pathway inhibitors to attenuate macrophage migration, can reduce macrophage tumor infiltration 43, these approaches may not be ideal as disease therapies. Many of the factors targeted by these approaches are not specific for monocyte/macrophages, including CCR2 and CSF1R, which are expressed on many other cell types 43. Systemic administration of drugs targeting these factors may therefore induce off target effects with unforeseen consequences. This is also a significant concern for macrophage reprogramming approaches, since the specificity of these approaches would rely on the monocyte/macrophage-specificity of the receptors or pathways targeted by anti-inflammatory factors or cytokine inhibitors after conventional systemic delivery.

### Nanomedicine-based approaches to modulate macrophage dysfunction

Nanomedicine, the medical application of nanotechnology, has emerged as a powerful platform to resolve issues associated with conventional therapeutics, including bioavailability, tissue specificity, and toxicity 45, 46. Significant progress has been made in the development of nanomedicine-based tools for disease diagnosis and treatment 45, including the use of nanoparticles to improve intranasal delivery of therapeutics for respiratory diseases or conditions 13, and multiple studies have examined the ability of nanomedicine therapies to reduce macrophage dysfunction in preclinical models of infectious or chronic inflammatory disease in attempts to increase efficiency and reduced side effects associated with conventional approaches (**Table 1**).

#### Nanomedicine approaches for macrophage targeting

Nanoparticles employed for macrophage targeting approaches can vary markedly in their origin (natural versus synthetic) and composition, but macrophage uptake generally occurs through one of two distinct pathways: non-specific phagocytosis (passive targeting) regulated by nanoparticle physical properties, or receptor-mediated endocytosis (active targeting). Passive targeting is usually responsible for macrophage uptake of unmodified nanoparticles of medium size (10~300 nm diameter), which include most extracellular vehicles (EVs) and liposomes. These nanoparticles primarily accumulate at infection or inflammation sites as a result of phagocytosis and/or macropinocytosis by monocyte/macrophage-lineage cells that are abundantly present at these sites 47, although *in vitro* studies indicate that some nanoparticles are also preferentially engulfed *via* clathrin- or caveolin-mediated endocytosis mechanisms 48. Both natural and synthetic nanoparticles can be modified to promote their active targeting to macrophages *via* different approaches that add macrophage-specific molecules to their surfaces 12. However, additional studies to identify surface markers associated with dysfunctional macrophages could improve the biodistribution and specificity of therapeutics targeted to this macrophage subpopulation. The following sections will discuss several possible nanomedicine-based approaches that might be employed to attenuate excessive macrophage activation responses associated with severe infectious and chronic disease.

#### Macrophage depletion with unmodified liposomes

At least two studies have now examined the efficacy of nanoparticle-mediated drug delivery to transiently deplete macrophages to attenuate dysfunctional macrophage responses (**Figure 1, Table 1**). These studies used synthetic liposomes, FDA-approved nanocarriers composed of one or more lipid bilayers surrounding a hollow core that can contain therapeutic cargoes 49. These particles lacked surface modifications and were primarily captured by macrophages and related phagocytic cells following their systemic administration due to their physical and chemical properties (passive targeting). Both these studies employed liposomes to increase intracellular delivery of clodronate, which induces macrophage depletion *via* disruption of the mitochondrial electron transport chain and cytosolic release of cytochrome C 50. Previous studies have revealed that liposome-mediated macrophage depletion has therapeutic capacity to lower cytokine production in autoimmune and infectious diseases, or animal model of these conditions, including rheumatoid arthritis and lipopolysaccharide (LPS)-induced sepsis 51-53. Macrophage depletion by clodronate-containing liposomes in rats with LPS-induced sepsis reduced hepatic IL-1β and TNF-α expression and plasma TNF-α levels 53. Similarly, clodronate-loaded liposomes suppressed IL-6 and MCP-1 expression, and the activation of downstream pro-inflammatory pathways including STAT3 and MAPK p38/ERK, mouse model of colon cancer induced by azoxymethane and dextran sodium sulfate treatment 52. A recent study in SARS-infected mice has also shown that antibody-mediated depletion of IMMs can effectively reduce lung lesions, cytokine levels (CCL2, TNF-α, and IL-6) and mortality, without altering viral load 54. These studies suggest the potential utility of macrophage depletion by liposomes, or other approaches, to suppress excess cytokine production and cytokine-associated pathology in severe infections or pro-inflammatory conditions. However, macrophage depletion may be beneficial only at the correct stage of infection. Potential adverse effects of systemic macrophage deletion, such as increased risk of infection and impairment of homeostatic macrophage functions in healthy tissues, are also not addressed by improving target specificity. Reprogramming dysfunctional macrophages with stem cells-derived EVs or synthetic nanoparticles may thus be more a more useful approach than total macrophage depletion when attempting to reduce pro-inflammatory cytokine production during excessive immune responses.

#### Macrophage reprogramming with unmodified EVs

Other studies examined the potential of unmodified EVs with anti-inflammatory properties to suppress macrophage dysfunction (**Figure 1**;** Table 1**). EVs are natural nanoparticles (~50-500 nm) that facilitate cell-to-cell communication, and are readily engulfed by circulating monocytes and tissue resident macrophages following their infusion into mouse models of human disease 55. Macrophage targeting efficacy of native EVs is partially determined by EV size, but may also be affected by the lipid and glycoprotein composition of their outer membrane, which can vary among EVs secreted by different parental cell types 56. EVs facilitate cell-to-cell communication by transporting endogenous bioactive molecules such proteins, nucleic acids, and lipids between cells 57. Mesenchymal stem cell-derived EVs (MSC-EVs) have been shown to potently reduce macrophage infiltration and cytokine release in models of acute lung injury (ALI) associated with severe pneumonia or sepsis 58-60. Systemic administration of human MSC-EVs reduced inflammatory cell influx and TNF-α expression in an *ex vivo* model in which perfused human lungs rejected for transplant were injured by *E. coli* exposure to induce severe bacterial pneumonia 59. Similarly, intratracheal MSC-EV administration reduced influenza virus replication and pro-inflammatory cytokine release (TNF-α and CXCL10) in the lungs of a pig model of influenza virus infection 60. Beneficial roles of MSC-EVs to decrease lung injury are at least partly due their ability to modulate respiratory macrophage phenotypes, as it has been reported that MSC-EVs can suppress pro-inflammatory M1-like and promote anti-inflammatory M2-like macrophage phenotypes in injured lungs 61. This effect appears to be regulated by endogenous microRNAs (*e.g.*, miR-21, miR-124) and proteins (*e.g.*, IL-10, TGF-β) shuttled by MSC-EVs 62. EV-mediated macrophage reprogramming may be superior to macrophage deletion, due to its reduced potential for negative short-term and long-term effects. MSC-EV therapeutic approaches may also offer a safety advantage due to their low potency for triggering adverse immune responses. Since EV therapeutic effects are regulated by their uptake efficiency and cargoes, EV therapeutic potency can be enhanced by loading them with therapeutic agents or modifying them to display macrophage-specific targeting molecules 63. Due to these advantages, stem cell-derived EVs have been investigated in early clinical studies of inflammatory and infectious diseases, and therapeutic effects of EVs from several MSC types are under investigation in current or planned COVID-19 trials 7.

#### Macrophage therapeutics using nanoparticles modified for target specificity

Functionalizing nanoparticle therapeutics with ligands or peptides that have high affinity for extracellular macrophage membrane factors can enhance their specific uptake to attenuate dysfunctional macrophage responses (**Figure 1**) 12. Synthetic nanocarriers (*e.g.*, solid-lipid, polymeric, or metallic nanoparticles) using this strategy have been developed for macrophage-targeted drug delivery in models of inflammatory and infectious diseases 12, 48. Several studies have employed different modification strategies to target nanoparticle drug delivery systems to macrophages. For example, nanoparticles functionalized with mannose were synthesized to promote nanoparticle uptake upon interaction with the macrophage mannose receptor, a PRR that recognizes specific carbohydrate patterns expressed on some pathogens 64. Macrophage-targeted delivery of TNF-α siRNA by these nanoparticles reduced TNF-α expression and colon damage in a mouse model of dextran sodium sulfate-induced colitis 64. In another study, alginate nanoparticles modified with tuftsin tetrapeptide (Thr-Lys-Pro-Arg), which specifically binds monocytes/macrophages, were used to deliver plasmid DNA encoding the anti-inflammatory cytokine IL-10. This approach was found to increase nanoparticle localization to inflammation sites, markedly increase the number of anti-inflammatory M2-like macrophages, and reduce joint damage and the expression of the pro-inflammatory cytokines IL-6, IL-1β, and TNF-α in the circulation and joints of an arthritic rat model 65. These studies indicate that nanoparticle-mediated, macrophage-selective drug delivery can attenuate pro-inflammatory macrophage responses and disease pathology, and has the potential to efficiently treat infected macrophages that may contribute to pathogen dissemination and macrophage dysfunction in some diseases. However, these synthetic nanoparticles may be more likely to trigger adverse immune responses than unmodified EVs and liposomes, and their biosafety needs to be carefully evaluated in clinical trials.

#### Therapeutic potential of virus-like particles

Virus-like particles (VLPs; 20~500 nm diameter), which contain viral coat proteins but lack viral genetic material, have recently been considered as nanoparticle platforms for drug delivery, imaging, and vaccine approaches 66, 67. Capsids from plant viruses are commonly used for VLP strategies to avoid potential adverse effects in the event the VLPs used contain residual genetic material 67. The effect of VLPs on macrophage-mediated immune responses is not well understood, but VLP treatment has been shown to have therapeutic effects in animal models of infection or chronic inflammation 68, 69. Nasal instillation of VLPs containing a synthetic single-stranded RNA and the capsid protein of the papaya mosaic virus was found to promote respiratory invasion by monocyte/macrophages, lymphocytes, and neutrophils and to enhance immune responses and survival upon subsequent challenge with influenza virus or *Streptococcus pneumoniae* 68. Similarly, mice injected with VLPs containing the cowpea mosaic virus capsid protein and an immunodominant peptide (p524) associated with type-1 diabetes were found to exhibit partial protection (delayed onset) from autoimmune diabetes 69. These studies suggest that VLPs may be a promising platform for prophylactic treatment approaches, but studies are required to demonstrate their ability to function as therapeutic agents that can limit macrophage dysfunction. Studies are also needed to address the stability and intrinsic immunogenicity of these particles, the latter of which is of particular concern for any approach seeking to attenuate pro-inflammatory responses.

## Conclusions and future perspectives

A growing body of evidence indicates the potential of macrophage-targeted nanomedicines to reduce excessive cytokine production during severe infection. We therefore believe that nanomedicine approaches designed to regulate macrophage-mediated inflammatory responses should receive greater consideration as alternative therapies for conditions where macrophage dysfunction has the potential to induce a cytokine storm and its associated pathological effects. To the best of our knowledge, studies have yet to examine the therapeutic potential of macrophage-targeted nanomedicines in animal models of COVID-19, the most recent disease where cytokine storms have been shown to play a central role in disease pathology and mortality. At minimum, we propose that EV and liposome studies should be performed using commercially available human ACE2 (hACE2) transgenic mice, which can serve as a model of human COVID-19 disease, to clarify whether nanomedicine-mediated macrophage depletion or attenuation of macrophage activation is beneficial in severe COVID-19 disease. Nanomedicine delivery approaches may also be useful to increase the targeted bioavailability or reduce potential side effects of anti-inflammatory small molecule drugs and antibody therapeutics currently under investigation for treatment of COVID-19 in clinical trials. It will be important to comprehensively evaluate the therapeutic effects of these and other potential nanomedicines using clinically relevant indicators, including, but not limited to, effects on survival, lung lesions, and cytokine profiles.

Some key issues should be considered before initiating such studies, however. Macrophage-targeted nanomedicines may need to be given at the correct stage of infection (*e.g.*, at or immediately prior to the hyperinflammatory phase), to have a beneficial effect, since suppression of the innate immune response in the early phase virus infection is likely to be harmful. Since cytokine storm development can be highly dynamic during severe infections, serial evaluation using a well-designed cytokine panel is necessary to determine the most effective therapeutic period and the specific effects of an intervention. Combination therapies that employ both nanomedicines and conventional antiviral drug delivery may also provide synergistic beneficial effects. Finally, it is also necessary to address the potential for increased risk of immune disorders and bacterial infections that may be associated with any strategy to deplete macrophages or attenuate their activity. Even if data from animal models proven to be promising, additional preclinical studies are required to address biosafety, mechanisms of action, and optimal administration routes and doses. Clinical translation will also require the development of nanomedicine manufacturing approaches with standard processes and quality control early in these studies. The massive degree of collaboration now ongoing across diverse disciplines and industries, offers hope for an unprecedent rate of development for new therapies and therapeutics to combat severe infectious diseases such as COVID-19. We believe that novel nanomedicine approaches are a worthy target of such efforts.

## Figures and Tables

**Table 1:** Examples of nanomedicine-based approaches to modulate macrophage dysfunction

Type of nanoparticles	Therapeutic cargos	Targeting mechanism	Impact on macrophages	Disease models	Therapeutic effects	Ref.
Human MSC-EVs	Endogenous miRNAs/Proteins	Passive targeting	Reprogramming	Mouse model of bacterial pneumonia	Improved survival and decreased neutrophils influx and cytokine in lungs	58
Human MSC-EVs	Endogenous miRNAs/Proteins	Passive targeting	Reprogramming	*Ex vivo* model of human bacterial pneumonia	Suppressed inflammatory cell influx and reduced TNF-α release	59
Swine MSC-EVs	Endogenous miRNAs/Proteins	Passive targeting	Reprogramming	Pig model of influenza virus infection	Reduced influenza virus replication and cytokines in the lungs	60
Human MSC-EVs	Endogenous miRNAs/Proteins	Passive targeting	Reprogramming	Mouse model of broncho-pulmonary dysplasia	Suppressed M1-like and promoted M2-like macrophages in the lungs	61
Liposomes	Clodronate	Passive targeting	Depletion	Rat model of sepsis	Reduced hepatic IL-1β/ TNF-α and plasma TNF-α	61
Liposomes	Clodronate	Passive targeting	Depletion	Mouse model of colon cancer	Suppressed IL-6/MCP-1 expression and STAT3 p38 MAPK/ERK signals in colon	52
TPP-PPM nanoparticles	TNF-α siRNA	Active targeting (Mannose receptor)	Reprogramming	Mouse model of colitis	Reduced TNF-α expression and colon damage	64
Tuftsin-alginate nanoparticles	Plasmid DNA encoding IL-10	Active targeting (Tuftsin peptide)	Reprogramming	Rat model of arthritis	Reduced cytokines IL-6, IL-1β, and TNF-α in blood and joints	65

**Notes:** mannosylated bioreducible cationic polymer (PPM), sodium triphosphate (TPP).

## References

[1] Chousterman BG, Swirski FK, Weber GF (2017). Cytokine storm and sepsis disease pathogenesis. Semin Immunopathol.

[2] Chen G, Wu D, Guo W, Cao Y, Huang D, Wang H (2020). Clinical and immunological features of severe and moderate coronavirus disease 2019. J Clin Invest.

[3] Mehta P, McAuley DF, Brown M, Sanchez E, Tattersall RS, Manson JJ (2020). COVID-19: consider cytokine storm syndromes and immunosuppression. Lancet.

[4] Moore JB, June CH (2020). Cytokine release syndrome in severe COVID-19. Science.

[5] Ye Q, Wang B, Mao J (2020). The pathogenesis and treatment of the `Cytokine Storm' in COVID-19. J Infect.

[6] Sarzi-Puttini P, Giorgi V, Sirotti S, Marotto D, Ardizzone S, Rizzardini G (2020). COVID-19, cytokines and immunosuppression: what can we learn from severe acute respiratory syndrome?. Clin Exp Rheumatol.

[7] Lythgoe MP, Middleton P (2020). Ongoing Clinical Trials for the Management of the COVID-19 Pandemic. Trends Pharmacol Sci.

[8] Byrne AJ, Mathie SA, Gregory LG, Lloyd CM (2015). Pulmonary macrophages: key players in the innate defence of the airways. Thorax.

[9] Labonte AC, Tosello-Trampont AC, Hahn YS (2014). The role of macrophage polarization in infectious and inflammatory diseases. Mol Cells.

[10] Mahajan S, Decker CE, Yang Z, Veis D, Mellins ED, Faccio R (2019). Plcgamma2/Tmem178 dependent pathway in myeloid cells modulates the pathogenesis of cytokine storm syndrome. J Autoimmun.

[11] Merad M, Martin JC (2020). Pathological inflammation in patients with COVID-19: a key role for monocytes and macrophages. Nat Rev Immunol.

[12] He H, Ghosh S, Yang H (2017). Nanomedicines for dysfunctional macrophage-associated diseases. J Control Release.

[13] Itani R, Tobaiqy M, Al Faraj A (2020). Optimizing use of theranostic nanoparticles as a life-saving strategy for treating COVID-19 patients. Theranostics.

[14] Speyer CL, Ward PA (2011). Role of endothelial chemokines and their receptors during inflammation. J Invest Surg.

[15] Medzhitov R (2007). Recognition of microorganisms and activation of the immune response. Nature.

[16] D'Elia RV, Harrison K, Oyston PC, Lukaszewski RA, Clark GC (2013). Targeting the "cytokine storm" for therapeutic benefit. Clin Vaccine Immunol.

[17] Zlotnik A, Yoshie O, Nomiyama H (2006). The chemokine and chemokine receptor superfamilies and their molecular evolution. Genome Biol.

[18] Ferrara JL, Abhyankar S, Gilliland DG (1993). Cytokine storm of graft-versus-host disease: a critical effector role for interleukin-1. Transplant Proc.

[19] Vaninov N (2020). In the eye of the COVID-19 cytokine storm. Nat Rev Immunol.

[20] Tisoncik JR, Korth MJ, Simmons CP, Farrar J, Martin TR, Katze MG (2012). Into the eye of the cytokine storm. Microbiol Mol Biol Rev.

[21] Jordan RE, Adab P, Cheng KK (2020). Covid-19: risk factors for severe disease and death. BMJ.

[22] Zabetakis I, Lordan R, Norton C, Tsoupras A (2020). COVID-19: The Inflammation Link and the Role of Nutrition in Potential Mitigation. Nutrients.

[23] Coperchini F, Chiovato L, Croce L, Magri F, Rotondi M (2020). The cytokine storm in COVID-19: An overview of the involvement of the chemokine/chemokine-receptor system. Cytokine Growth Factor Rev.

[24] Koo SJ, Garg NJ (2019). Metabolic programming of macrophage functions and pathogens control. Redox Biol.

[25] Shapouri-Moghaddam A, Mohammadian S, Vazini H, Taghadosi M, Esmaeili SA, Mardani F (2018). Macrophage plasticity, polarization, and function in health and disease. J Cell Physiol.

[26] Aderem A, Underhill DM (1999). Mechanisms of phagocytosis in macrophages. Annu Rev Immunol.

[27] Jakubzick CV, Randolph GJ, Henson PM (2017). Monocyte differentiation and antigen-presenting functions. Nat Rev Immunol.

[28] Zhang X, Mosser DM (2008). Macrophage activation by endogenous danger signals. J Pathol.

[29] Kyriazopoulou E, Leventogiannis K, Norrby-Teglund A, Dimopoulos G, Pantazi A, Orfanos SE (2017). Macrophage activation-like syndrome: an immunological entity associated with rapid progression to death in sepsis. BMC Med.

[30] Arango Duque G, Descoteaux A (2014). Macrophage cytokines: involvement in immunity and infectious diseases. Front Immunol.

[31] Giamarellos-Bourboulis EJ, Netea MG, Rovina N, Akinosoglou K, Antoniadou A, Antonakos N (2020). Complex Immune Dysregulation in COVID-19 Patients with Severe Respiratory Failure. Cell Host Microbe.

[32] Ruan Q, Yang K, Wang W, Jiang L, Song J (2020). Clinical predictors of mortality due to COVID-19 based on an analysis of data of 150 patients from Wuhan, China. Intensive Care Med.

[33] Kang S, Tanaka T, Narazaki M, Kishimoto T (2019). Targeting Interleukin-6 Signaling in Clinic. Immunity.

[34] Channappanavar R, Perlman S (2017). Pathogenic human coronavirus infections: causes and consequences of cytokine storm and immunopathology. Semin Immunopathol.

[35] Carsana L, Sonzogni A, Nasr A, Rossi R, Pellegrinelli A, Zerbi P (2020). Pulmonary post-mortem findings in a large series of COVID-19 cases from Northern Italy. medRxiv. 2020.

[36] Nikitina E, Larionova I, Choinzonov E, Kzhyshkowska J (2018). Monocytes and Macrophages as Viral Targets and Reservoirs. Int J Mol Sci.

[37] Keidar S, Strizevsky A, Raz A, Gamliel-Lazarovich A (2007). ACE2 activity is increased in monocyte-derived macrophages from prehypertensive subjects. Nephrol Dial Transplant.

[38] Chen y, Feng Z, Diao B, Wang R, Wang G, Wang C (2020). The Novel Severe Acute Respiratory Syndrome Coronavirus 2 (SARS-CoV-2) Directly Decimates Human Spleens and Lymph Nodes. medRxiv. 2020.

[39] Mogensen TH (2009). Pathogen recognition and inflammatory signaling in innate immune defenses. Clin Microbiol Rev.

[40] Karakike E, Giamarellos-Bourboulis EJ (2019). Macrophage Activation-Like Syndrome: A Distinct Entity Leading to Early Death in Sepsis. Front Immunol.

[41] Peterson KR, Cottam MA, Kennedy AJ, Hasty AH (2018). Macrophage-Targeted Therapeutics for Metabolic Disease. Trends Pharmacol Sci.

[42] Ponzoni M, Pastorino F, Di Paolo D, Perri P, Brignole C (2018). Targeting Macrophages as a Potential Therapeutic Intervention: Impact on Inflammatory Diseases and Cancer. Int J Mol Sci.

[43] Cassetta L, Pollard JW (2018). Targeting macrophages: therapeutic approaches in cancer. Nat Rev Drug Discov.

[44] Schultze JL (2016). Reprogramming of macrophages-new opportunities for therapeutic targeting. Curr Opin Pharmacol.

[45] Chung BL, Toth MJ, Kamaly N, Sei YJ, Becraft J, Mulder WJ (2015). Nanomedicines for Endothelial Disorders. Nano Today.

[46] Kamaly N, He JC, Ausiello DA, Farokhzad OC (2016). Nanomedicines for renal disease: current status and future applications. Nat Rev Nephrol.

[47] Weissleder R, Nahrendorf M, Pittet MJ (2014). Imaging macrophages with nanoparticles. Nat Mater.

[48] Colino CI, Lanao JM, Gutierrez-Millan C (2020). Targeting of Hepatic Macrophages by Therapeutic Nanoparticles. Front Immunol.

[49] Allen TM, Cullis PR (2013). Liposomal drug delivery systems: from concept to clinical applications. Adv Drug Deliv Rev.

[50] van Rooijen N, van Kesteren-Hendrikx E (2002). Clodronate liposomes: perspectives in research and therapeutics. J Liposome Res.

[51] Vanniasinghe AS, Bender V, Manolios N (2009). The potential of liposomal drug delivery for the treatment of inflammatory arthritis. Semin Arthritis Rheum.

[52] Bader JE, Enos RT, Velazquez KT, Carson MS, Nagarkatti M, Nagarkatti PS (2018). Macrophage depletion using clodronate liposomes decreases tumorigenesis and alters gut microbiota in the AOM/DSS mouse model of colon cancer. Am J Physiol Gastrointest Liver Physiol.

[53] Sturm E, Havinga R, Baller JF, Wolters H, van Rooijen N, Kamps JA (2005). Kupffer cell depletion with liposomal clodronate prevents suppression of Ntcp expression in endotoxin-treated rats. J Hepatol.

[54] Channappanavar R, Fehr AR, Vijay R, Mack M, Zhao J, Meyerholz DK (2016). Dysregulated Type I Interferon and Inflammatory Monocyte-Macrophage Responses Cause Lethal Pneumonia in SARS-CoV-Infected Mice. Cell Host Microbe.

[55] Imai T, Takahashi Y, Nishikawa M, Kato K, Morishita M, Yamashita T (2015). Macrophage-dependent clearance of systemically administered B16BL6-derived exosomes from the blood circulation in mice. J Extracell Vesicles.

[56] Mulcahy LA, Pink RC, Carter DR (2014). Routes and mechanisms of extracellular vesicle uptake. J Extracell Vesicles.

[57] Phinney DG, Pittenger MF (2017). Concise Review: MSC-Derived Exosomes for Cell-Free Therapy. Stem Cells.

[58] Monsel A, Zhu YG, Gennai S, Hao Q, Hu S, Rouby JJ (2015). Therapeutic Effects of Human Mesenchymal Stem Cell-derived Microvesicles in Severe Pneumonia in Mice. Am J Respir Crit Care Med.

[59] Park J, Kim S, Lim H, Liu A, Hu S, Lee J (2019). Therapeutic effects of human mesenchymal stem cell microvesicles in an ex vivo perfused human lung injured with severe E. coli pneumonia. Thorax.

[60] Khatri M, Richardson LA, Meulia T (2018). Mesenchymal stem cell-derived extracellular vesicles attenuate influenza virus-induced acute lung injury in a pig model. Stem Cell Res Ther.

[61] Willis GR, Fernandez-Gonzalez A, Anastas J, Vitali SH, Liu X, Ericsson M (2018). Mesenchymal Stromal Cell Exosomes Ameliorate Experimental Bronchopulmonary Dysplasia and Restore Lung Function through Macrophage Immunomodulation. Am J Respir Crit Care Med.

[62] Lai P, Weng J, Guo L, Chen X, Du X (2019). Novel insights into MSC-EVs therapy for immune diseases. Biomark Res.

[63] Armstrong JP, Holme MN, Stevens MM (2017). Re-Engineering Extracellular Vesicles as Smart Nanoscale Therapeutics. ACS Nano.

[64] Xiao B, Laroui H, Ayyadurai S, Viennois E, Charania MA, Zhang Y (2013). Mannosylated bioreducible nanoparticle-mediated macrophage-specific TNF-alpha RNA interference for IBD therapy. Biomaterials.

[65] Jain S, Tran TH, Amiji M (2015). Macrophage repolarization with targeted alginate nanoparticles containing IL-10 plasmid DNA for the treatment of experimental arthritis. Biomaterials.

[66] Koudelka KJ, Pitek AS, Manchester M, Steinmetz NF (2015). Virus-Based Nanoparticles as Versatile Nanomachines. Annu Rev Virol.

[67] Jeevanandam J, Pal K, Danquah MK (2019). Virus-like nanoparticles as a novel delivery tool in gene therapy. Biochimie.

[68] Mathieu C, Rioux G, Dumas MC, Leclerc D (2013). Induction of innate immunity in lungs with virus-like nanoparticles leads to protection against influenza and Streptococcus pneumoniae challenge. Nanomedicine.

[69] Zampieri R, Brozzetti A, Pericolini E, Bartoloni E, Gabrielli E, Roselletti E (2020). Prevention and treatment of autoimmune diseases with plant virus nanoparticles. Science Advances.

